# Expression pattern, subcellular localization of *Aspergillus oryzae* ergosterol synthases, and their effects on ergosterol and fatty acid metabolism

**DOI:** 10.1128/aem.02273-24

**Published:** 2025-03-04

**Authors:** Xueqin Tian, Kunhai Qin, Yunhong Deng, Pinghong Xue, Chaozheng Huang, Shaofang Liu, Zhihong Hu

**Affiliations:** 1 College of Life Sciences, Jiangxi Science and Technology Normal University 177505 , Nanchang, China; 2 Jiangxi Key Laboratory of Natural Microbial Medicine Research 177505 , Nanchang, China; Chalmers tekniska hogskola AB, Gothenburg, Sweden

**Keywords:** *Aspergillus oryzae*, ergosterol, subcellular localization, fatty acid

## Abstract

Ergosterol is an important component of fungal cell membranes and participates in the regulation of its fluidity, permeability, and material transport. In our study, we found that the ergosterol biosynthesis pathway in *Aspergillus oryzae* is more complex than that in *Saccharomyces cerevisiae*, involving 49 enzymes and 25 reactions. In this study, we systematically analyzed the expression pattern and subcellular localization of *A. oryzae* ergosterol synthases and their effects on ergosterol and fatty acid metabolism. The results showed that ergosterol synthase genes were distributed across the eight chromosomes of the *A. oryzae* genome and were differentially expressed at different growth times. Subcellular localization analysis revealed that the mevalonate biosynthesis-related enzymes were localized in the cytoplasm, mitochondria, and peroxisomes; farnesyl pyrophosphate biosynthesis-related enzymes were mainly localized in the cytoplasm, peroxisomes, and mitochondria; while several ergosterol biosynthesis-related enzymes were localized in the endoplasmic reticulum and lipid droplets. Overexpression (OE) of these enzymes affected both ergosterol and fatty acid contents in *A. oryzae*. The ergosterol content was the highest in the *AoHmgB*-OE strain and the lowest in the *AoHmgA*-OE strain, while the fatty acid content was the highest in the *AoErg26B*-OE strain and the lowest in the *AoErg26A*-OE strain. Moreover, the ergosterol content was significantly increased in the *AoHmgB*/*AoErg7B*-, *AoHmgB*/*AoErg9*-, and *AoErg1*/*AoErg7B*-co-OE strains compared to the single gene OE strains. Our study results may offer a scientific foundation for genetic engineering research focusing on lipid metabolism in *A. oryzae* and related fungi, thereby facilitating the creation of fungal strains with enhanced ergosterol production.

**IMPORTANCE:**

Ergosterol, an important fungal cell membrane component, participates in the regulation of membrane fluidity, permeability, and material transport. Previous studies have demonstrated that the ergosterol biosynthesis pathway in *Aspergillus oryzae* exhibits greater complexity compared to that in *Saccharomyces cerevisiae*; nonetheless, research on the ergosterol biosynthesis pathway in *A. oryzae* remains limited. In this study, we determined the expression pattern and subcellular localization of ergosterol biosynthesis-related enzymes in *A. oryzae*. Additionally, we assessed the effects of the overexpression (OE) of ergosterol biosynthesis-related genes on ergosterol and fatty acid contents in *A. oryzae*. Therefore, our study may provide a scientific basis for genetic engineering research on lipid metabolism in *A. oryzae* and other fungal species.

## INTRODUCTION

Sterols, a large group of naturally occurring hydroxylated steroid macromolecules, are the key components of plasma and mitochondrial membranes (1). In cell membranes, sphingolipid-associated sterols are involved in the formation of microdomains called lipid rafts, which are essential for anchoring various biological molecules, such as receptors, efflux pumps, nutrient transporters, and sodium and potassium pumps (2, 3). Sterols uphold the structural integrity of lipid rafts, thereby influencing cellular functions, stress responses, and adaptation (2). Sterols can be classified into three types based on their source: animal sterols (cholesterol), phytosterols (campesterol, stigmasterol, sitosterol, oat sterols, and spinach sterols, and fungisterol (ergosterol) (4). Ergosterol is specifically present in fungal cell membranes and can thus serve as a biomarker to assess fungal biomass (5). Additionally, the ergosterol biosynthesis pathway can serve as a therapeutic target for the treatment of fungal infections in humans, animals, and plants (6).

Research on fungal species, especially yeast, has greatly progressed our understanding of the ergosterol biosynthesis pathway. In *Saccharomyces cerevisiae*, the ergosterol biosynthesis pathway involves 24 reactions, catalyzed by 25 enzymes (including two isozymes, Hmg1 and Hmg2) (7). The pathway can be divided into three phases based on the intermediate products: mevalonate biosynthesis, farnesyl pyrophosphate (farnesyl-PP) biosynthesis, and ergosterol biosynthesis (7). Mevalonate biosynthesis involves successive catalytic reactions by Erg10 (thiolase), Erg13 (hydroxymethylglutaryl-CoA synthase), and Hmg1/2 (Hmg-CoA reductases). Erg10 catalyzes the condensation of two acetyl coenzyme A (acetyl-CoA) molecules to form acetoacetyl-CoA; Erg13 catalyzes the addition of a third acetyl-CoA molecule to acetoacetyl-CoA, yielding 3-hydroxy-3-methylglutaryl-CoA (Hmg-CoA); lastly, Hmg1/2 catalyzes the reduction of Hmg-CoA to mevalonate (8). Farnesyl pyrophosphate (farnesyl-PP) biosynthesis from mevalonate is successively catalyzed by Erg12, Erg8, Erg19, Idi1, and Erg20 (9). Farnesyl-PP is an essential intermediate metabolite and is used for the synthesis of ubiquinone, dolichol, heme, sterols, and prenylated proteins (10, 11). Therefore, the inhibition of the farnesyl-PP biosynthesis pathway can lead to cell death. Ergosterol production from farnesyl-PP is catalyzed by a total of 16 enzymes (8). It begins with the conversion of farnesyl-PP to squalene, followed by squalene cyclization to lanosterol, which is then converted to ergosterol through a series of reactions (9). The ergosterol biosynthesis pathway has been studied extensively (7, 12). The mevalonate biosynthetic pathway, which is crucial for the synthesis of sterols and terpenoids, is conserved across all eukaryotes, including plants and animals. Farnesyl-PP and ergosterol biosynthetic pathways, on the other hand, are specific to fungi and are not found in other eukaryotic organisms. In *S. cerevisiae*, the biosynthesis processes of mevalonate, farnesyl-PP, and ergosterol mainly take place in the vacuole/mitochondria, vacuole, and endoplasmic reticulum (ER), respectively (13).

Cellular lipid metabolism is a complex network of pathways, which are precisely regulated and coordinated to maintain lipid homeostasis and cell function (13, 14). Acetyl-CoA is a final product of fatty acid beta-oxidation, and it serves as a substrate for both ergosterol and fatty acid biosyntheses (7, 15). These reports suggest that ergosterol and fatty acid metabolism may have a closely regulated network. For instance, HMG-CoA reductase inhibition increases fatty acid synthesis in keratinocytes (16). Additionally, the fatty acid decarboxylase in *Jeotgalicoccus* species also catalyzes the conversion of mevalonate into 3-methyl-3-butan-1-ol (17). Moreover, the overexpression (OE) or knockdown of mevalonate diphosphate decarboxylase (involved in the ergosterol biosynthesis pathway) affects both ergosterol and fatty acid contents in *A. oryzae* (18).

*A. oryzae* (koji mold) is a filamentous fungus that has been utilized for the production of traditional Asian fermented foods, including sauce, miso, and sake, for millennia. Recently, it has also found applications in the commercial production of enzymes. In our previous study, we conducted bioinformatics analysis to identify the genes involved in ergosterol biosynthesis in *A. oryzae* (19). Our results indicated that the ergosterol biosynthesis pathway is conserved in *A. oryzae*, consistent with the well-documented pathway in fungi, as evidenced by the extensive research on the genes involved in ergosterol synthesis and their regulatory roles. *A. oryzae* has multiple paralogues of almost all the ergosterol biosynthesis genes, which are not observed obviously in *S. cerevisiae*. For example, *AoErg10* has six paralogues and *AoHmg* and *AoErg20* have five paralogues each. The enzymes catalyzing the 24 reactions of the ergosterol biosynthesis pathway in *A. oryzae* are encoded by 49 genes, indicating the presence of several isozymes. These results suggest that compared to *S. cerevisiae*, *A. oryzae* has a more complex ergosterol biosynthesis pathway. However, research on the function of ergosterol biosynthesis gene paralogues of *A. oryzae* is limited except for our research about six paralogues of *AoErg10* (20). In this study, we systematically analyzed the expression pattern and subcellular localization of all ergosterol synthases in *A. oryzae* and investigated the effects of their OE on fatty acid metabolism. This represents the first systematic research on ergosterol synthases in filamentous fungi, thereby, the results of this study may serve as the foundation for future gene-related studies.

## RESULTS

### Expression patterns of ergosterol biosynthesis genes in *A. oryzae*

**Fig 1:**
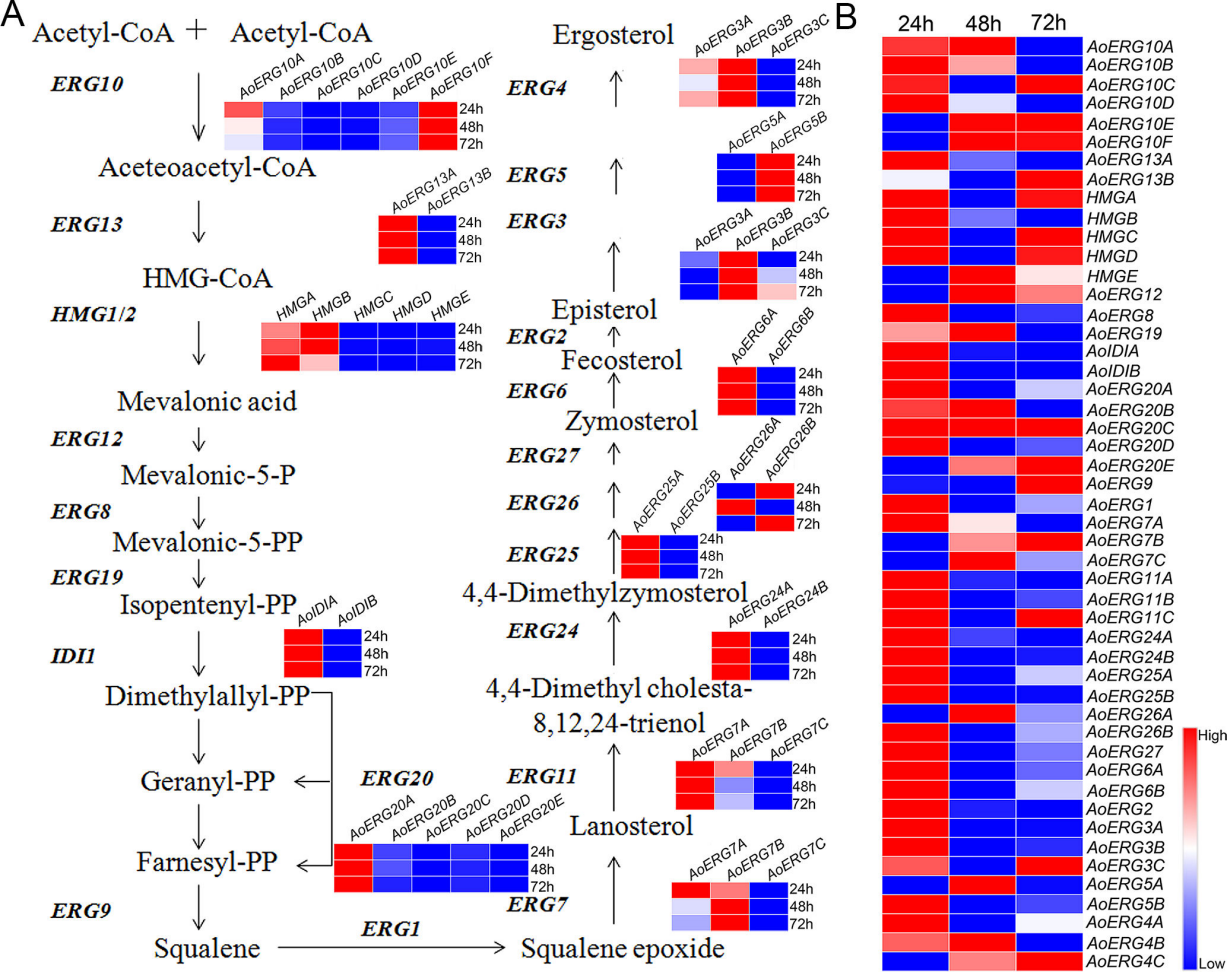
Expression profile comparison of ergosterol biosynthesis genes. (**A**) Comparison of multiple homologous ergosterol biosynthesis gene expression levels at the same growth time. (**B**) Comparison of ergosterol biosynthesis gene expression levels at different growth times.

Ergosterol biosynthesis involves 24 reactions, which are catalyzed by 49 enzymes in *A. oryzae* (19). The 49 genes responsible for ergosterol biosynthesis are randomly distributed across the eight chromosomes of *A. oryzae*, as illustrated in Fig. S1. We systematically analyzed the expression pattern of all the ergosterol biosynthesis genes at 24, 48, and 72 h growth periods using the transcriptome data obtained in our previous study (21). The heatmap in Fig. 1A shows the expression of the paralogous ergosterol biosynthesis genes during different growth times. *AoHmgA* and *AoHmgB* were expressed during all growth periods (Fig. 1A). Figure 1B shows the expression of all the ergosterol biosynthesis genes at 24, 48, and 72 h growth periods. As shown in Fig. 1B, most ergosterol biosynthesis genes exhibited high expression at the 24 h growth stage. However, the expression levels of AoErg10C, AoErg13B, AoHmgA, and AoHmgC, among others, declined after the 48 h growth stage, whereas the expression of AoErg10A, AoErg10B, AoErg10D, and AoErg13A decreased after the 72 h growth period. The fpkm value of ergosterol biosynthesis genes at different growth times is shown in Table S1. These results indicate that the cell may require more lipids at the early growth stage to expand rapidly and form the cell membrane.

### Subcellular localization of ergosterol synthases in *A. oryzae*

Our previous study showed that the subcellular localization and function of AoErg10s and AoErg19 showed differences in *A. oryzae* compared with other species (20, 22). This study focused on elucidating the subcellular localization of all ergosterol synthases in *A. oryzae*. For this, ergosterol synthase-encoding genes were fused with *DsRed* fluorescent protein and transformed into *A. oryzae*. We observed all transformants under a fluorescence microscope. The results indicated that all recombinant proteins, except AoIdi1B, were expressed successfully (Fig. S2 to S5). The AoIdi1B was fused at both the C- and N-terminals of *DsRed* and *GFP*; however, its fluorescence was not observed in *A. oryzae*. The subcellular localization of all the ergosterol synthases was further investigated based on their role in mevalonate, farnesyl-PP, and ergosterol biosyntheses.

### Mevalonate biosynthesis-related enzymes were localized in the peroxisome

**Fig 2:**
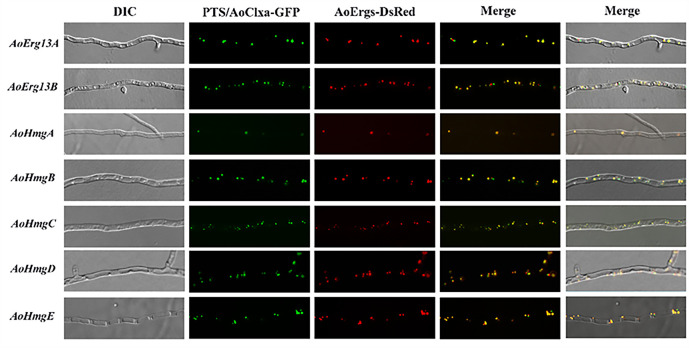
Subcellular localization of *A. oryzae* mevalonate biosynthetic enzymes. Co-localization of AoErg13A/B-*DsRed* and AoHmgA–E-*DsRed* with peroxisome and endoplasmic reticulum markers. The *A.oryzae* Δ*pyrG*Δ*His* strain was co-transformed with AoErg13A/B*-DsRed* or AoHmgB–E-*DsRed* with *GFP*-PTSI or AoClxA-*GFP* vectors. Left to right: differential interference contrast (DIC), fluorescent images of GFP, fluorescent images of DsRed, merged images of GFP and DsRed, and merged images of DIC, GFP, and DsRed.

Mevalonic acid biosynthesis is catalyzed by AoErg10s, AoErg13s, and AoHmgs. Our previous study revealed that AoErg10s are localized in the cytoplasm, mitochondria, or peroxisomes (20). Colocalization analysis using peroxisome and ER-localization markers (GFP-PTSI and AoClax-GFP, respectively) revealed that AoErg13A/B and AoHmgB–E were colocalized with GFP-PTSI, a peroxisome localization marker, while AoHmgA was colocalized with AoClax-GFP (Fig. 2) but not GFP-PTSI (Fig. S6). These results indicate that mevalonic acid biosynthesis may occur primarily in the peroxisomes of *A. oryzae*.

### Farnesyl-PP biosynthesis-related enzymes were localized in the peroxisome, cytoplasm, and vacuoles

**Fig 3:**
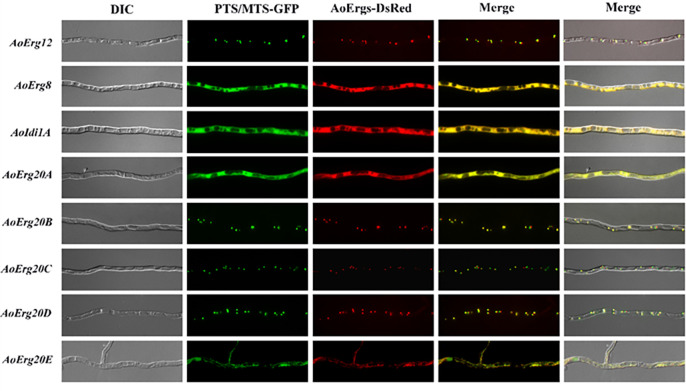
Subcellular localization of farnesyl-PP biosynthetic enzymes. The mycelium transformed with AoErg8-*DsRed*, AoIdi1A-*DsRed*, and AoErg20A-*DsRed* were found to be located in the cytoplasm and colocalized with *GFP* without target signals. The mycelium transformed with AoErg12-*DsRed* and AoErg20B–D-*DsRed* were colocalized with PTS-*GFP*. The mycelium transformed with AoErg20E-*DsRed* was colocalized with MTS-*GFP* vectors. Left to right: DIC, fluorescent images of GFP, fluorescent images of DsRed, merged images of GFP and DsRed, and merged images of DIC, GFP, and DsRed.

Farnesyl-PP biosynthesis in *A. oryzae* involves five reactions, which are sequentially catalyzed by AoErg12, AoErg8, AoErg19, AoIdi1s, and AoErg20s. Our previous study revealed that AoErg19 is localized in vacuoles (22). In this study, we found that AoErg12 was localized in peroxisomes, while AoErg8 and AoIdi1A were localized in the cytoplasm (Fig. 3). However, the fluorescence of C/N-tagged *DsRed* or *GFP* fused AoIdi1B could not be observed in *A. oryzae*. AoErg20 has five paralogues (AoErg20A–E), which catalyze the conversion of dimethylallyl-PP to geranyl-PP and the conversion of geranyl-PP to farnesyl-PP. The results demonstrated that AoErg20A is localized in the cytoplasm, while AoErg20B–D is found in peroxisomes, and AoErg20E resides in mitochondria. These results indicate that farnesyl-PP biosynthesis may occur sequentially in peroxisomes, cytoplasm, and vacuoles.

### Enzymes involved in ergosterol biosynthesis are localized in the ER and/or within lipid droplets

**Fig 4:**
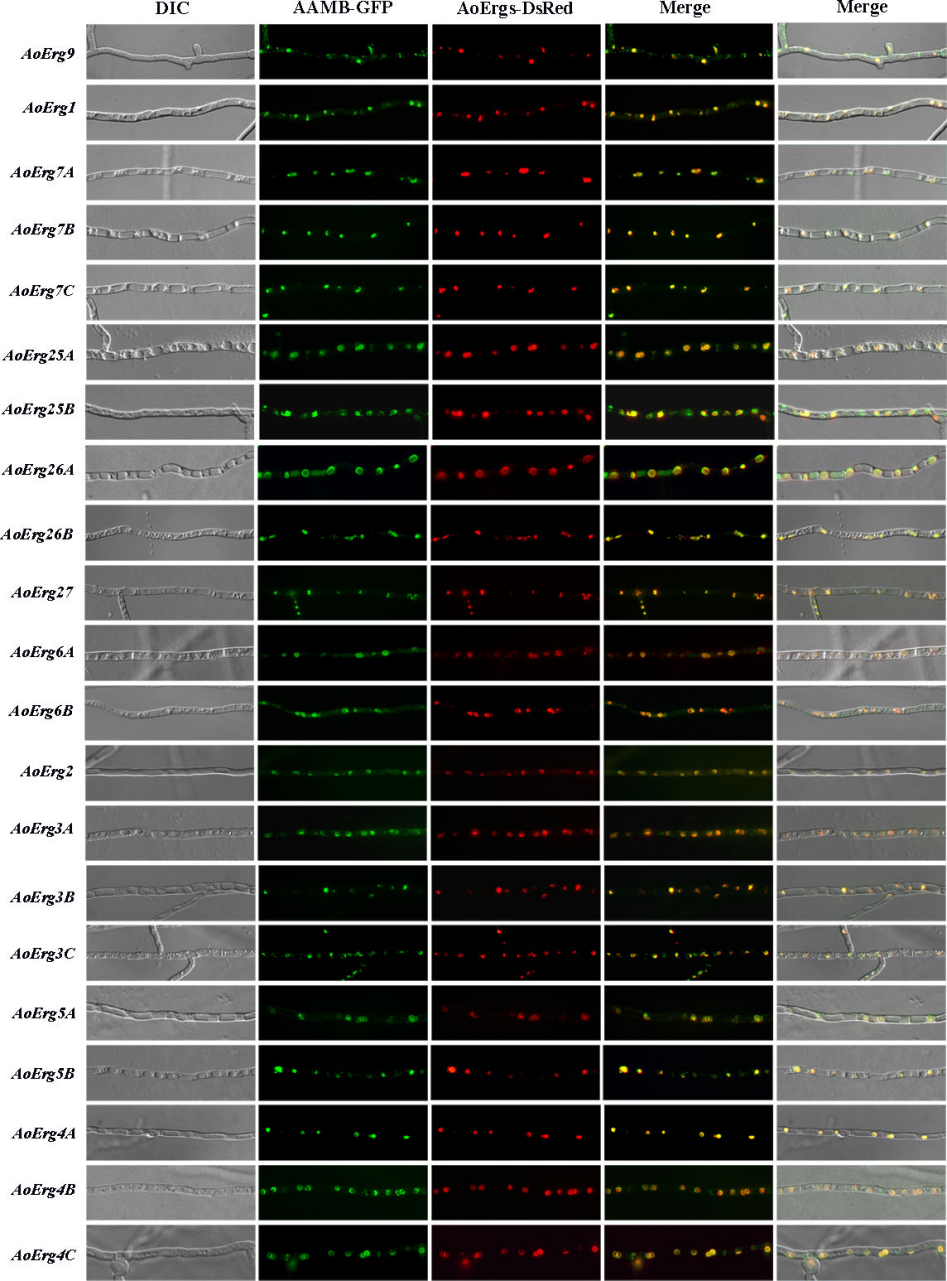
Subcellular localization of enzymes from squalene to ergosterol biosynthesis. The mycelium of *A.oryzae* Δ*pyrG*Δ*His* was co-transformed with AoErgs-*DsRed* and AAMB-*GFP* vectors for fluorescence observation. Left to right: differential interference contrast, fluorescent images of GFP, DsRed, merged images of GFP and DsRed, and merged images of GFP, DsRed, and differential interference contrast.

Ergosterol synthesis from squalene is catalyzed by 16 enzymes in *A. oryzae*, which are encoded by 26 genes. All the intermediates of this pathway are lipid-soluble, suggesting that the enzymes involved in ergosterol synthesis are localized in the ER or lipid drops. Consistently, our results revealed that all 26 enzymes involved in the transformation of squalene to ergosterol were localized in the ER (Fig. S7) and/or lipid drops (Fig. 4). These results suggest that ergosterol biosynthesis may occur primarily in the ER and/or lipid drops.

### Phenotypes of the ergosterol synthase overexpression strains

**Fig 5:**
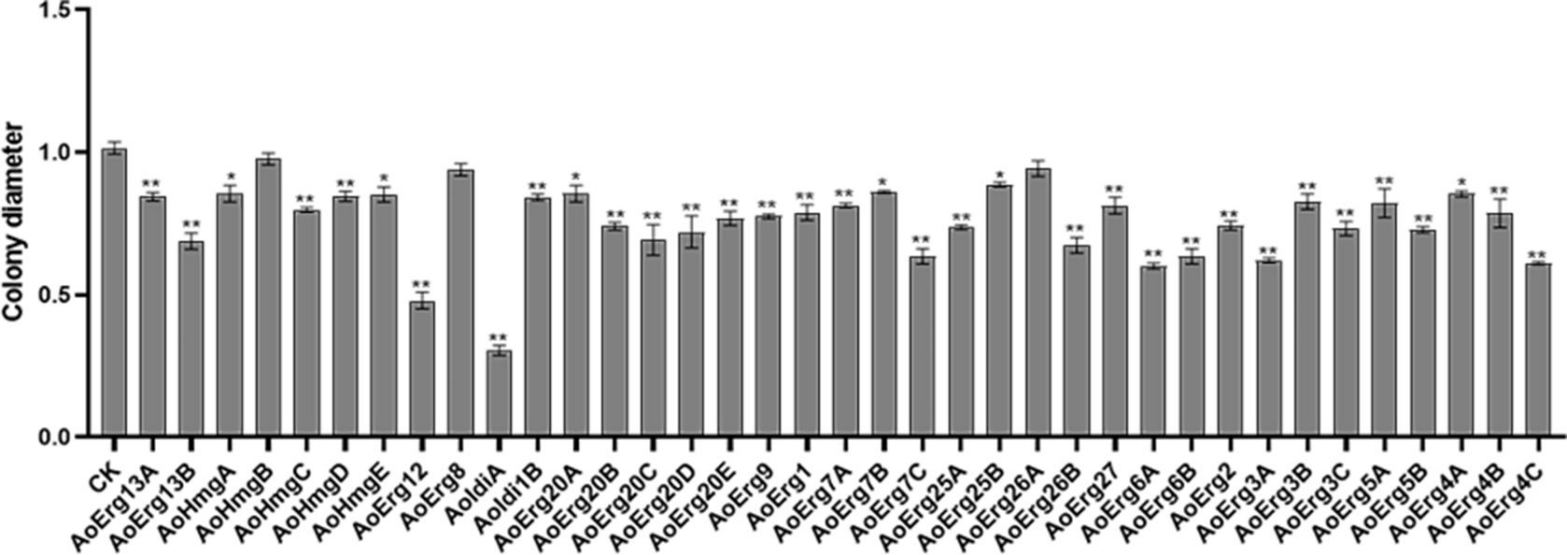
Phenotypes of ergosterol synthase overexpression strains. The relative diameters of colonies of different ergosterol synthase overexpression strains after 72 h cultivation on CD + His medium.

All the ergosterol synthase OE strains were cultured on CD + His medium, and their phenotypes were observed (Fig. S8). As shown in Fig. 5, all the transgenic strains, except *AoHmgB*-, *AoErg8*-, and *AoErg26A*-OE strains, showed delayed growth, as indicated by their smaller colony diameters compared to that of the control strain. Among these, *AoErg12*- and *AoIdi1A*-OE strains showed significantly reduced growth. These results indicate that the OE of the majority of ergosterol synthases may delay the growth of *A. oryzae*.

### Ergosterol and fatty acid contents in the ergosterol synthase overexpression strains

**Fig 6:**
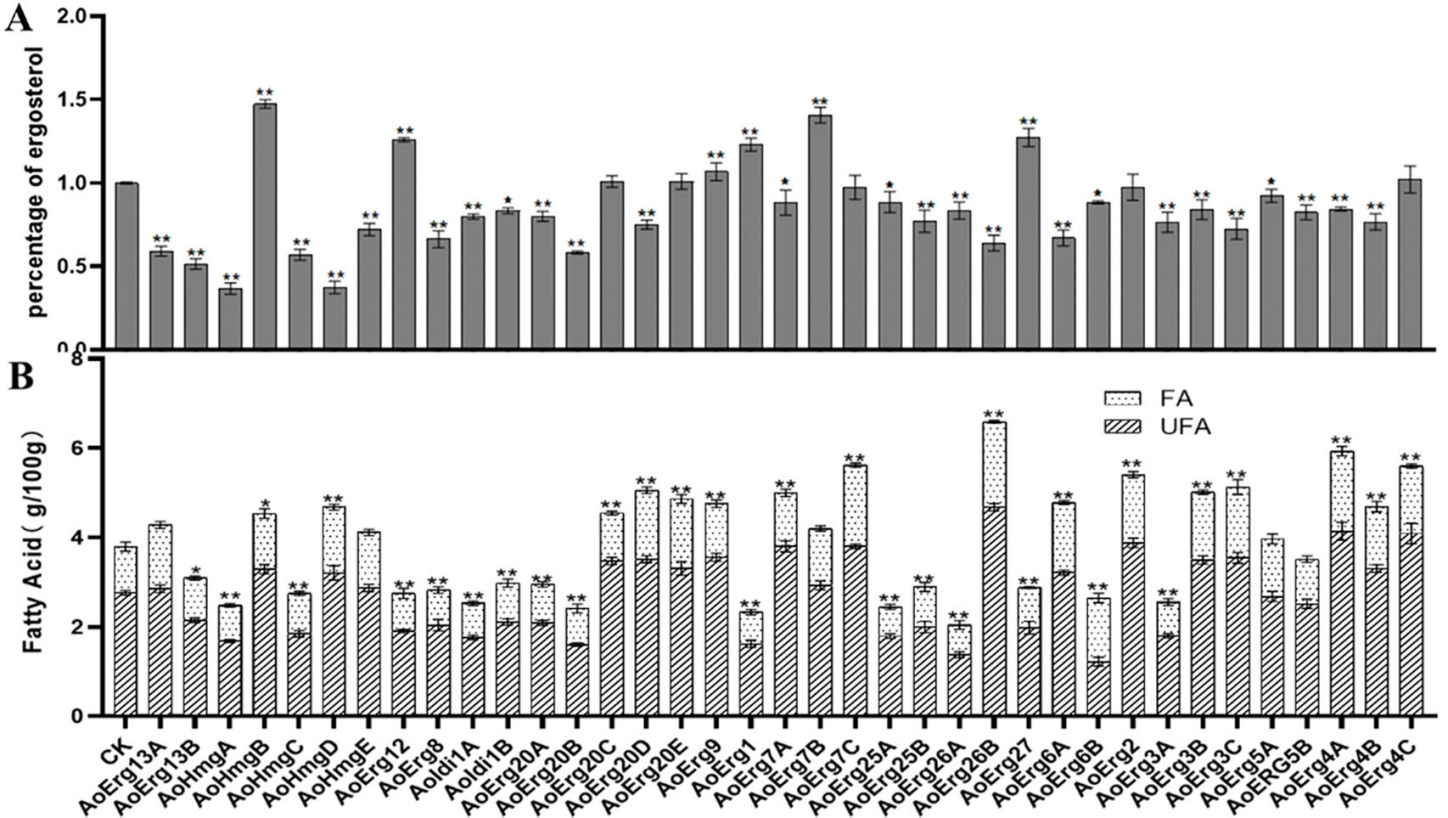
Ergosterol and fatty acid contents in ergosterol synthase overexpression strains. (**A**) The relative ergosterol content in ergosterol synthetase overexpression strains. (**B**) The fatty acid contents in ergosterol synthase overexpression strains. CK: the strain of *A.oryzae* Δ*pyrG* Δ*His* transformed with pEX2B vector, FA: saturated fatty acid, UFA: unsaturated fatty acid.

Given the importance of ergosterol in natural product synthesis and its role as a substrate for biosynthesis, we assessed the ergosterol and fatty acid content in all ergosterol synthase OE strains (Fig. 6). The results showed that ergosterol content was unaltered in the *AoErg20C*-, *AoErg20E*-, *AoErg7C*-, *AoErg2*-, and *AoErg4C*-OE strains; increased in the *AoHmgB*-, *AoErg12*-, *AoErg9*-, *AoErg1*-, *AoErg7B*-, and *AoErg27*-OE strains; and decreased in the remaining OE strains (Fig. 6A). Interestingly, ergosterol content was highly increased in the *AoHmgB-*OE strain and highly decreased in the *AoHmgA*-OE strain (Fig. 6A). Additionally, our results showed that fatty acid content was increased in the *AoErg2*-, *AoErg3B/C*-, *AoErg4A–C*-, *AoErg5A*-, *AoErg6A*-, *AoErg7A–C*-, *AoErg9*-, *AoErg13A*-, *AoErg20C–E*-, *AoErg26B*-, and *AoHmgB/D/E*-OE strains but decreased in the remaining 17 OE strains (Fig. 6B). The specific values are shown in Table S2. These results indicate that the OE of ergosterol synthases can affect the contents of both ergosterol and fatty acids in *A. oryzae*.

### Effects of co-overexpression of ergosterol synthases on ergosterol content in *A*. oryzae

**Fig 7:**
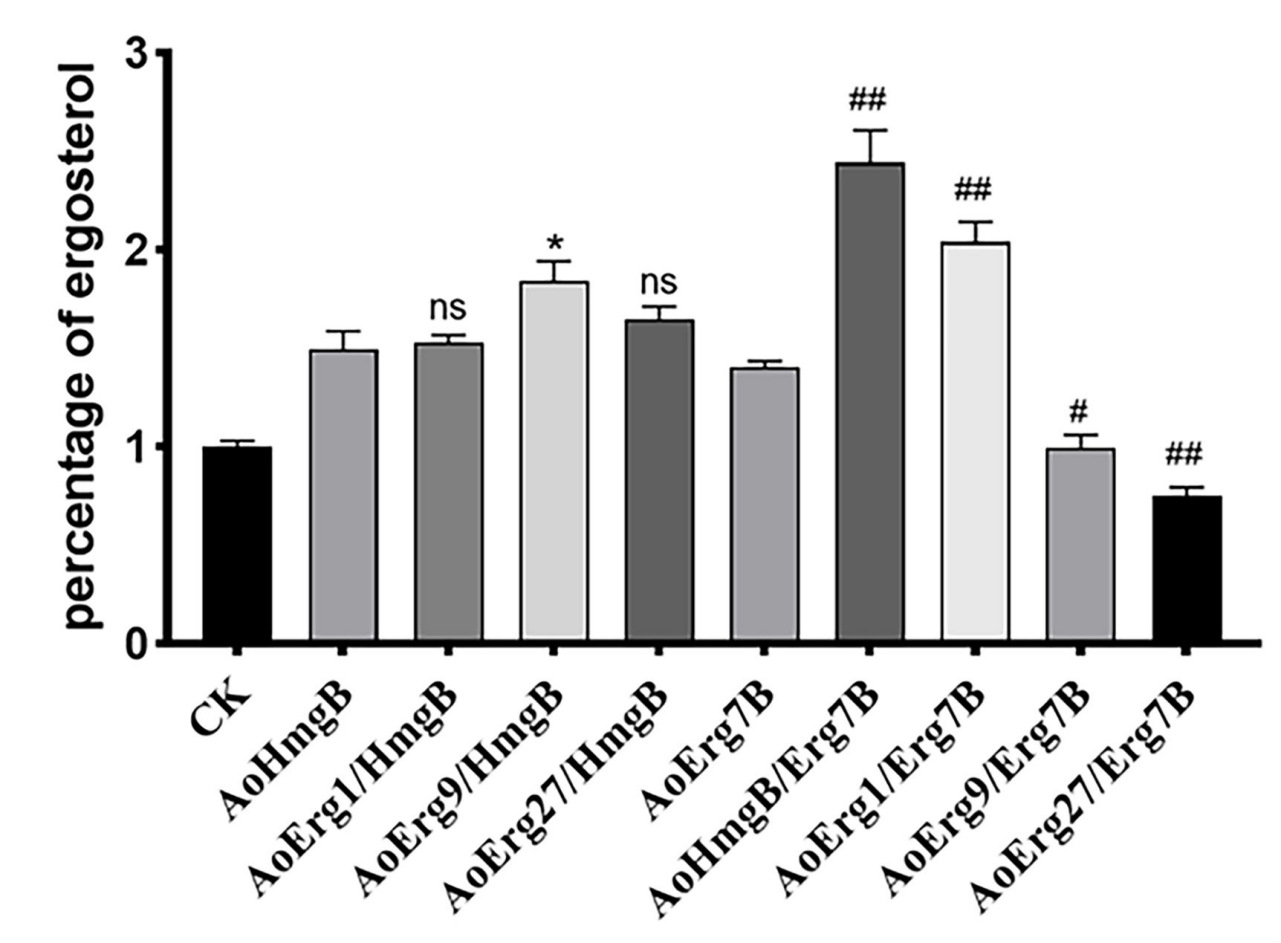
The relative ergosterol contents in combination with over-expression of ergosterol synthases strains. CK: the strain of *A. oryzae* Δ*pyrG*Δ*His* transformed with pEX2B vector. Asterisks denote significant differences (*P* < 0.05).

Ergosterol is a precursor for steroid synthesis. In this study, we used co-OE of specific ergosterol synthases to increase ergosterol content in *A. oryzae*. The *AoHmgB*- and *AoErg7B*-OE strains were selected as the background to overexpress the *AoErg9*, *AoErg1*, and *AoErg27* genes. The co-OE of these genes was validated by fluorescence microscopy (Fig. S9). The results showed that the ergosterol content was significantly increased in the *AoHmgB*/*AoErg7B*, *AoErg9*/*AoHmgB*, and *AoErg1*/*AoErg7B* co-OE strains and significantly decreased in the *AoErg9*/*AoErg7B* and *AoErg27*/*AoErg7B* compared to the background strains (Fig. 7). These results indicate that co-OE of specific ergosterol synthases can increase or decrease ergosterol content in *A. oryzae* compared to single gene OE.

### Transcriptome analysis of *A*. *oryzae*

**Fig 8:**
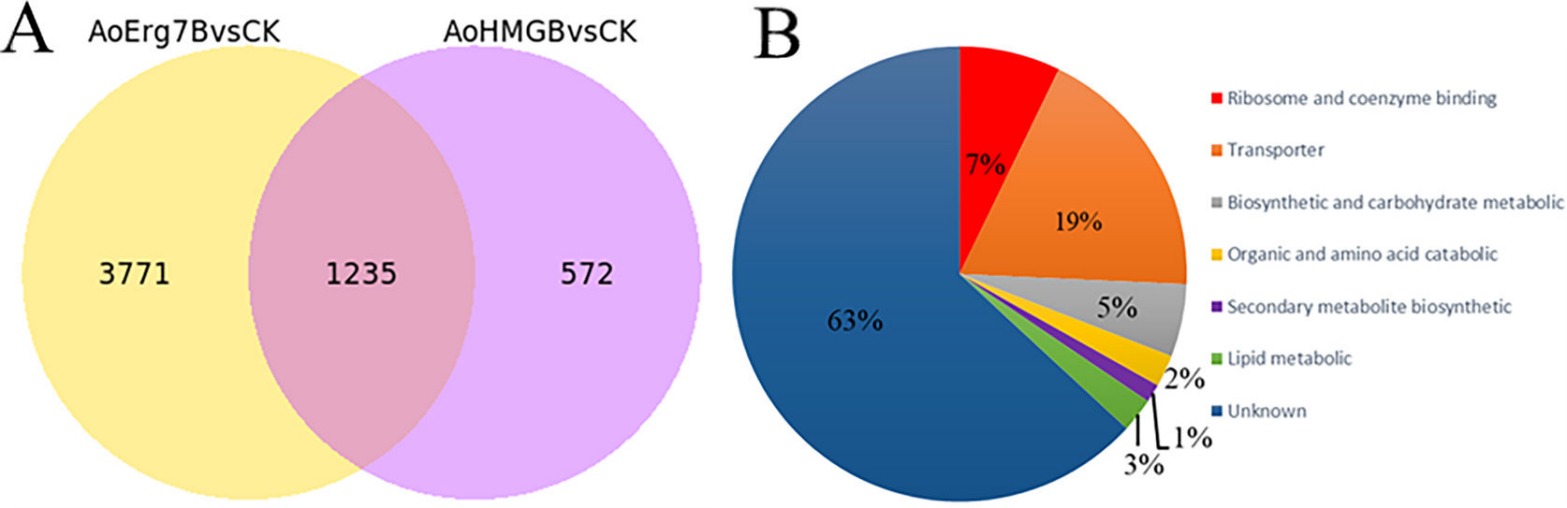
Analysis of DEGs between AoErg7B-OE treatment and AoHmgB-OE treatment. (**A**) Venn diagram of genes regulated by both AoErg7B-OE (right) treatment and AoHmgB-OE (left) treatment. (**B**) The classification of DEGs regulated by both AoErg7B and AoHmgB overexpression strains.

To investigate the relationship between ergosterol and fatty acid biosyntheses, the *AoHmgB*- and *AoErg7B*-OE strains were subjected to transcriptome analysis. The overview data of transcriptome are shown in Table S3. Compared to the control, the AoErg7B-OE strain had 5,006 differentially expressed genes (DEGs), while the AoHmgB-OE strain had 1,807 DEGs. Of these, 1,235 DEGs were common in both *AoErg7B*- and *AoHmgB*-OE strains (Fig. 8A). Among the common DEGs, 63% were unknown, 19% were transporter-encoding genes, 7% were ribosome/coenzyme-binding protein-encoding genes, and 5% were lipid metabolism-related genes (Fig. 8B). The Gene Ontology (GO) classifications of DEGs in AoHmgB and AoErg7B are shown in Table S4 and S5. The putative functions of the differentially expressed lipid metabolism-related proteins are listed in Table 1. Additionally, the changes in the expression of ergosterol biosynthesis-related genes in the *AoErg7B*- and *AoHmgB*-OE strains are listed in Table S6.

**TABLE 1:** Putative functions of the differentially expressed fatty acid metabolism-related genes in the *AoHmgB*- and *AoErg7B*-OE strains

Accession number	Putative production encoded by the gene	Fold change in gene expression	
		*AoHmgB*-OE	*AoErg7B*-OE
EIT82406.1	Acyl-CoA oxidase	4.42	−1.32
EIT79189.1	3-Hydroxyacyl-CoA dehydrogenase	3.33	−3.35
EIT73836.1	Alcohol dehydrogenase, class V	−3.06	−6.06
EIT72694.1	Aldehyde dehydrogenase	1.22	2.05
EIT80755.1	Alcohol dehydrogenase, class III	1.18	−2.55

## DISCUSSION

Ergosterol is not only essential for fungal growth and reproduction. It is also important for stress adaptation and can be used as a direct precursor for steroidal drug production (23). Identifying genes and enzymes involved in ergosterol biosynthesis is crucial. On the one hand, ergosterol and some of its biosynthetic intermediates are important metabolites of great economic value. On the other hand, it may lay the foundation for the discovery of ergosterol biosynthesis enzyme inhibitors, which may be applied in agricultural and medical fungal prevention and treatment. Previous studies showed the ergosterol biosynthesis pathway in filamentous fungi is much more complex than that in *S. cerevisiae* (19, 24–26). This study systematically analyzed the expression patterns, and subcellular localization of ergosterol synthases in *A. oryzae*, as well as their impacts on ergosterol and fatty acid metabolism.

### Expression and subcellular localization of ergosterol biosynthesis enzymes

Based on the ergosterol biosynthesis pathway in *S. cerevisiae*, we identified 49 ergosterol biosynthesis-related genes in *A. oryzae*, including several paralogous and homologous genes (7), which were randomly distributed across its eight chromosomes. The expression pattern analysis showed that the majority of ergosterol biosynthesis-related genes were highly expressed after 24 h of growth; however, the expression of some genes decreased after 48 h (AoErg10C) and 72 h (AoErg13A) of growth. We hypothesized that during the early growth phase, cells grow rapidly, requiring high amounts of lipids to synthesize cell membranes. Therefore, the expression of these genes may reduce after 48 h or 72 h of growth. Moreover, some ergosterol biosynthesis-related genes showed increased expression at 72 h, indicating that they may be associated with conidia formation.

We have categorized the research on ergosterol localization in *A. oryzae* into three sections based on the properties of intermediate products, demonstrating that mevalonate synthase is primarily localized in peroxisomes, while the synthase of the farnesyl-pp module is predominantly found in peroxisomes, cytoplasm, and vesicles. The remaining ergosterol synthase is mainly localized in the endoplasmic reticulum and lipid droplets. Interestingly, ergosterol content in the AoHmgB-OE strain was increased while in the AoHmgA-OE strain was decreased (Fig. 6A). The explanation is that the different subcellular localization may play different roles in the function of the enzyme. AoHmgB-E is localized in the peroxisome, whereas AoHmgA is localized to the endoplasmic reticulum. In *Magnaporthe oryzae*, there are two Acetoacetyl-CoA acetyltransferases (MoAcat1 and MoAcat2), which show different expression patterns and subcellular localizations and play different diverse functions (27). In our previous study, we also identified that the different localization of six thiolases (AoErg10s) play different functions in *A. oryzae* (20). Similarly, in *S. cerevisiae*, reports have indicated that HMG1/2 serves as the rate-limiting enzyme for the mevalonic acid (MVA) pathway. Therefore, we deduced that AoHmgB might function as the rate-limiting enzyme in the ergosterol synthesis pathway of *A. oryzae*.

### Effect of ergosterol biosynthesis overexpression on ergosterol and lipid metabolism in *A. oryzae*

Ergosterol plays a crucial role in fungal cell membranes, influencing their fluidity and, consequently, cell growth. Thus, ergosterol content is strictly regulated to appropriate levels by feedback regulation of ergosterol synthesizing activities at the transcriptional, translational, and posttranslational levels (28). Our research indicated that only a few ergosterol biosynthesis enzyme OE-strains possess the capacity to modify ergosterol content, either by augmenting or diminishing it. OE of a single enzyme can increase by no more than 1.5 times that of control. Indeed, the combined application of OE strategies has led to a significant increase in ergosterol content, reaching a maximum of approximately 2.3 times that of the control. Besides, some gene OE decreased ergosterol content which may be explained by our previous study that OE of AoErg19 resulted in decreased ergosterol content by feedback regulation (22).

As described, the regulation of ergosterol content is stringently maintained at optimal levels to ensure cellular viability. Therefore, the OE of ergosterol biosynthesis genes in specific organelles, leading to ergosterol accumulation within these organelles, could represent a potent strategy for further elevating ergosterol content. This method has been successfully applied in *S. cerevisiae*. For example, dual metabolic engineering targeting cytoplasmic and mitochondrial acetyl-CoA utilization emerges as an effective means to boost isoprene production in *S. cerevisiae* (29); and dual metabolic engineering of the MVA pathway in both mitochondria and cytoplasm is also a powerful way to increase linalool production in *S. cerevisiae* (30). Therefore, accurately determining the subcellular localization of ergosterol biosynthesis is crucial for genetically engineering it to enhance ergosterol or intermediate production.

As mentioned, the ergosterol biosynthesis pathway is initiated by the condensation of two acetyl-CoA molecules (6), acetyl-CoA is also a final product of fatty acid beta-oxidation and substrate for fatty acid biosynthesis or elongation (15). Therefore, the ergosterol biosynthesis pathway and fatty acid biosynthesis may have a closely regulated network. Indeed, in this study, we found OE of ergosterol biosynthesis can also affect fatty acid contents. Notably, the total fatty acid content was the highest in the AoErg26B-OE strain and the lowest in the AoErg26A-OE strain. This result indicated that Erg26A and Erg26B may have distinct roles in fatty acid synthesis, depending on the ergosterol content depletion. Therefore, our findings demonstrated that the various paralogues in *A. oryzae* may exhibit distinct functions, further highlighting the complexity of the ergosterol biosynthesis pathway.

Transcriptome analysis was conducted to determine the effects of AoHmgB and AoErg7B-OE on the gene expression profile of *A. oryzae*. The results showed that 1,235 DEGs were common between the AoErg7B- and AoHmgB-OE strains, of which the majority were unannotated genes and five were associated with lipid metabolism. Additional research and inquiry are justified to explore further the possible function of these five genes, particularly scrutinizing if they are indeed the key focuses through which the ergosterol biosynthesis process is controlled and if they also have a crucial role in the regulation of fatty acid biosynthesis. Notable consequences for the further enhancement of ergosterol and fatty acid levels in production.

## MATERIALS AND METHODS

### Preparation of cDNA libraries and RNA sequencing

According to the manufacturer’s instructions, total RNA was extracted from 0.5 g of mycelia using a fungal RNA kit (Omega Bio-tek, Norcross, GA, USA) with the addition of an RNase-free DNase I treatment (Omega). The concentration of RNA was determined utilizing a NanoDrop ND-1000 spectrophotometer (Thermo Scientific, Wilmington, DE, USA), while the integrity of the RNA was analyzed employing a Bioanalyzer 2100 (Agilent Technologies, Palo Alto, CA, USA). In order to ensure reliability and reproducibility, each pooled RNA sample was prepared from an equal amount of RNA from three individual cultures for cDNA library construction. Following the enrichment of the pooled total RNA using oligo (dT) magnetic beads, the mRNA was subjected to fragmentation in a buffer solution at 94°C for a period of 5 min. First-strand cDNA was synthesized using random hexamer primers with mRNA fragments serving as templates. A second-strand cDNA was synthesized using DNA polymerase I and RNase H. Following end repair and adaptor ligation, the products were amplified by PCR and purified using a QIAquick PCR purification kit (Qiagen) to generate a cDNA library. Subsequently, the constructed libraries were subjected to sequencing on an Illumina HiSeq 2500 platform (Illumina, USA).

### Analysis of data

The raw data underwent stringent quality control measures, ensuring the creation of high-quality data sets with clean reads. This process involved the elimination of reads with low quality, adaptor sequences, and those containing more than 10% N’s (ambiguous bases). The resultant clean reads, with Q30 scores exceeding 85%, were then aligned to the *A. oryzae* genome using the Tophat (v2.0.7) software. In parallel, the Q30, GC content, and sequence duplication rate of the clean data were also calculated, with the GC content being a measure of the ratio of guanine (G) and cytosine (C) nucleotides in the sequence. The screening of strains for DEGs was conducted using edgeR software. The significance threshold for differential expression was set at padj <0.05 and log_2_(foldchange) >2. The Cluster Profiler software was employed to conduct GO function enrichment and Kyoto Encyclopedia of Genes and Genomes (KEGG) pathway enrichment analyses of differential gene sets.

### Statistical analysis

Results were analyzed via one-way analysis of variance and Student’s *t*-test, utilizing GraphPad Prism 8 software (San Diego, CA, USA). The former was employed for the significance analysis of multiple comparisons, while the latter was used for the comparison of two different groups. Each treatment was replicated three times, and data are presented as mean ± SD.

### Strains and growth conditions

The *A. oryzae* RIB40 Δ*pyrG* Δ*His* trophic deficiency strain (donated by Dr. Van-Tuan Tran; Vietnam National University–University of Science, Hanoi, Vietnam) was used as the receptor strain for fungal transformation (31). The DPY medium (2% sucrose, 1% peptone, 0.5% yeast extract, 0.5% KH_2_PO_4_, 0.05% MgSO_4_, and 1.6% agar; pH 5.5) supplemented with 0.1% uridine, 0.1% uracil, and 0.1% histidine was used for fungal cultivation. *A. oryzae* was cultured in the DPY medium at 30°C for 3 days. The culture was then mixed with sterile distilled water and filtered using a sterile filter paper. The concentration of the spores was determined using a hemocytometer. The plasmid vectors used for the transfection of *A. oryzae* were stored in *Escherichia coli* Trans-T1 (32) and *Agrobacterium tumefaciens* AGL1 (33). *E. coli* was cultured in Luria–Bertani (LB) medium supplemented with kanamycin at 37°C, while *A. tumefaciens* was cultured in LB medium supplemented with rifampicin at 28°C.

### Gene overexpression

The binary vector *pEX2B* (34) was used to overexpress ergosterol synthase genes. The *pEX2B* vector was linearized using AflII, and the target DNA fragments were cloned to the restriction site using a one-step cloning kit (Vazyme Biotech Co., Ltd., China). The primers specifically designed for target DNA amplification are listed in Table S7. All the recombinant vectors (*pEX2B-AoErgs-DsRed*) were transfected into *A. tumefaciens* AGL1 cells, which were then used for *A. tumefaciens*-mediated transformation of *A. oryzae* RIB40 Δ*pyrG* Δ*His* strain, as described in our previous publication (35). To verify the successful OE of genes, we employed fluorescence microscopy to observe and confirm the expression in over three transformants for each gene and selected the strain with the most pronounced fluorescence for further analysis. Monoclonal transformants were collected and cultured for further analysis.

### Subcellular localization

The *pEX2B* vector with the *DsRed* reporter gene was used as the control to detect the subcellular localization of ergosterol synthases. The binary vector *pEX1-His*, incorporating *hisB* as a selective marker, histidine for screening, and *GFP* as a reporter gene, was developed to precisely ascertain the subcellular localization of ergosterol synthases, considering their pivotal roles in mevalonate, farnesyl-PP, and ergosterol biosynthetic pathways. Seven common organelle-targeting signal vectors were constructed in this study. The SRL amino acids and Golgi-targeting sequences were fused to the C-terminus of *GFP* to generate different organelle-targeting signals, which were then cloned into *pEX1-His* vector to construct pEX1-His-GFP-PTSI (peroxisome-targeting vector) and pEX1-His-GFP-Aogos1 vectors (36, 37). Similarly, the targeting sequences of the nucleus (H2B), mitochondria (MTS), vacuoles (Aovam), ER (Aoclxa), and lipid droplets (AAMB) were fused to the N-terminal of *GFP*, which were subsequently cloned into pEX1-His vector to construct pEX1-His-H2B-GFP, pEX1-His-MTS-GFP, pEX1-His-Aovam-GFP, pEX1-His-Aoclxa-GFP, pEX1-His-AAMB-GFP vectors, respectively (24, 38–42). The recombinant vectors containing the targeted signals were transformed into the ergosterol synthase OE strains, and the transformed strains were observed under a fluorescence microscope (Fig. S10).

### Measurement of ergosterol and fatty acid contents

Ergosterol and fatty acid contents were measured as described in a previous report (18, 20, 22), with two minor modifications. First, *A. oryzae* mycelium was cultured in the presence of exogenous histidine. Second, total fat extraction was conducted using methyl tert-butyl ether instead of ether. Three biological replicates were performed for all measured strains and analyzed by GraphPad Prism 8 software.

### Gene co-overexpression

The *pEX1-His-GFP* vectors were used to study the effects of gene co-OE of ergosterol synthases in *A. oryzae*. The vectors were linearized with XhoI, and the target gene sequences of *AoErg1*, *AoErg9*, *AoErg27*, and *AoErg7B* were cloned to the restriction site using the one-step cloning kit to construct the pEX1-His-DNA-GFP vectors. The specific primers used for target gene amplification are shown in Table S8. The constructed plasmids were stored in *E. coli* and transfected into *A. tumefaciens*.

## Data Availability

Sequencing data were deposited in the NCBI SRA database under Bioproject accession no. PRJNA1188350 and BioSample accession no. SAMN44832574.

## SUPPLEMENTAL MATERIAL

Supplemental material aem.02273-24-s0001.docx Figures S1 to S10; Tables S1 to S8.

ASM does not own the copyrights to Supplemental Material that may be linked to, or accessed through, an article. The authors have granted ASM a non-exclusive, world-wide license to publish the Supplemental Material files. Please contact the corresponding author directly for reuse.
