# Supplementary material for: Expression pattern, subcellular localization of Aspergillus oryzae ergosterol synthases, and their effects on ergosterol and fatty acid metabolism
Source: Appl Environ Microbiol. 2025 Mar 4;91(4):e02273-24. doi: 10.1128/aem.02273-24 (PMC12016551; doi:10.1128/aem.02273-24)
Supplement: Supplemental material — Figures S1 to S10; Tables S1 to S8. [file aem.02273-24-s0001.docx]

# Supplementary material

**Supplementary figure S1.** Diagram of ergosterol synthesis gene distribution in the chromosome.

##
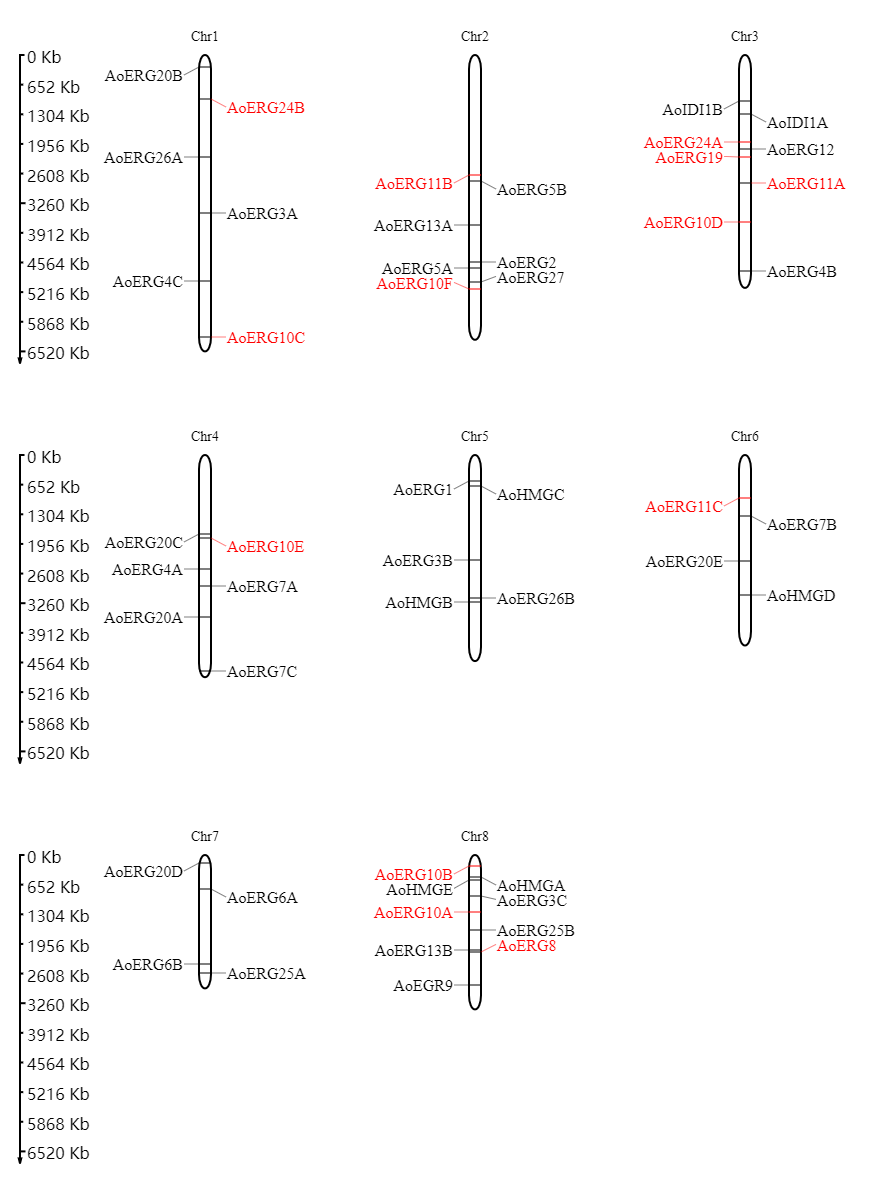


## Supplementary figure S2. Images of fluorescence-labeled mevalonate biosynthetic enzyme in corresponding over-expressing strains. Left to right: differential interference contrast (DIC), fluorescent images of DsRed, merged images of DIC and DsRed.


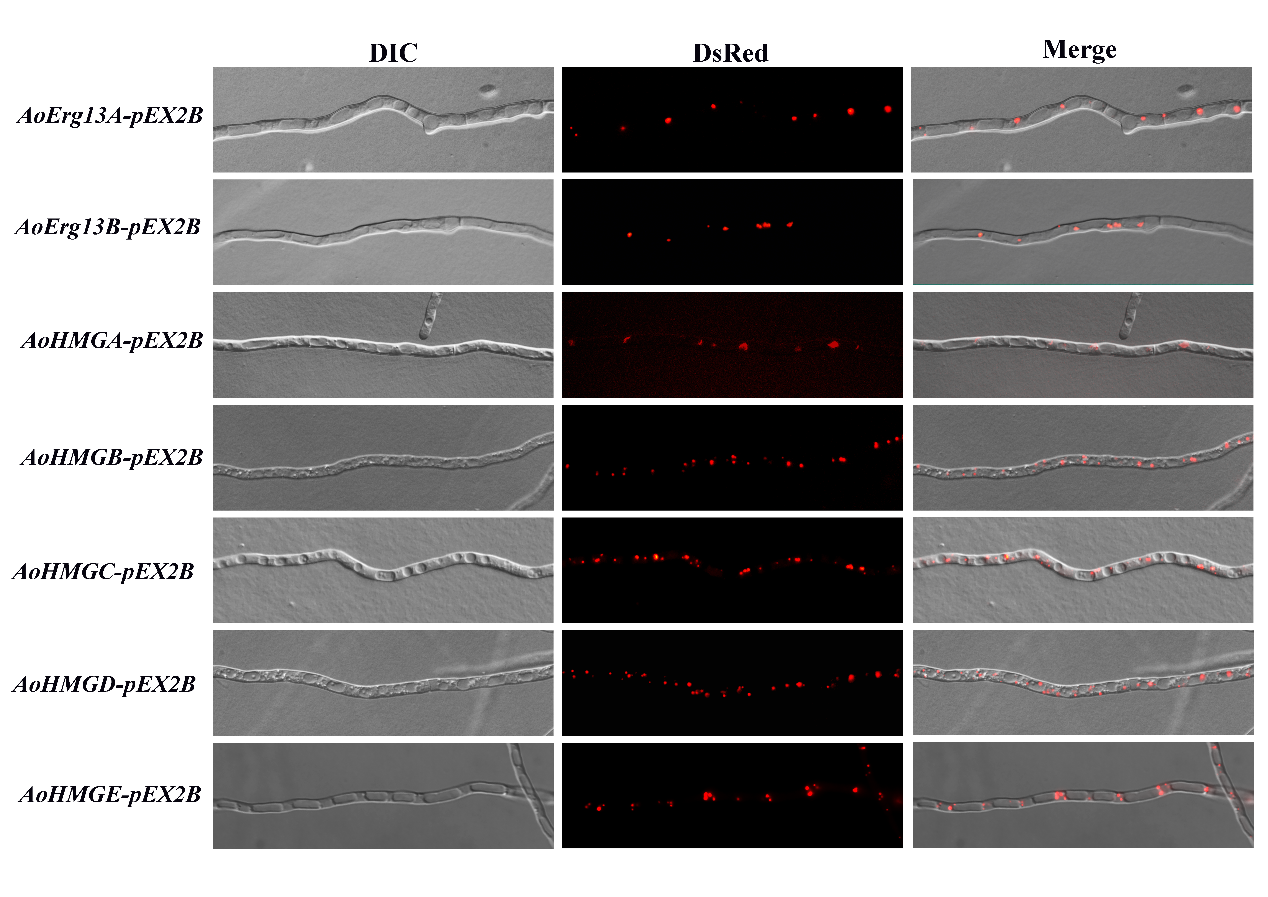


**Supplementary figure S3.** Images of fluorescence-labeled farnesyl pyrophosphate biosynthetic enzyme in corresponding over-expressing strains. Left to right: differential interference contrast (DIC), fluorescent images of DsRed, merged images of DIC and DsRed.


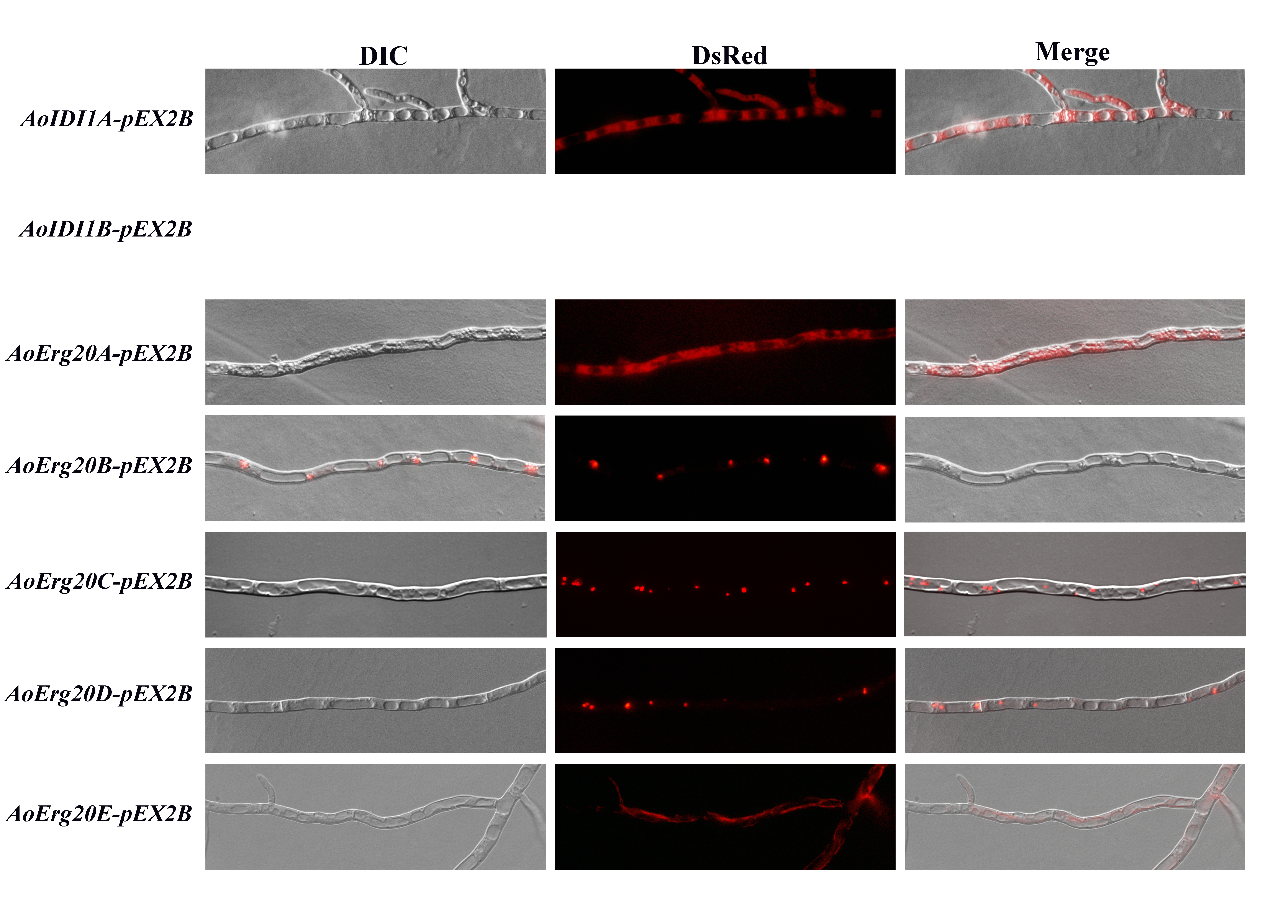


**Supplementary figure S4-5.** Images of ergosterol biosynthetic enzyme over-expressing strains under fluorescence microscope. Left to right: differential interference contrast (DIC), fluorescent images of DsRed, merged images of DIC and DsRed.


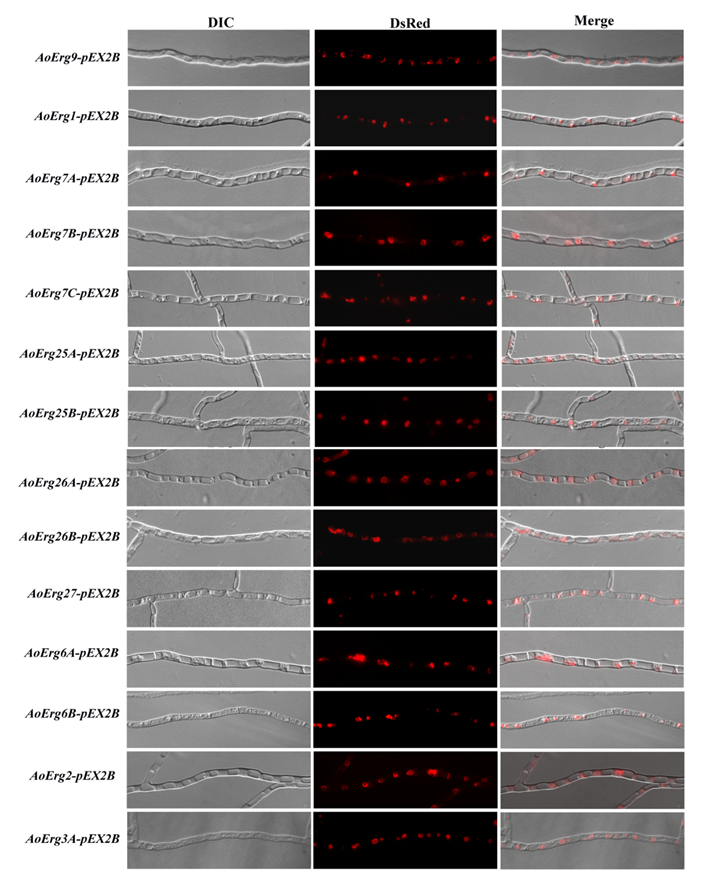


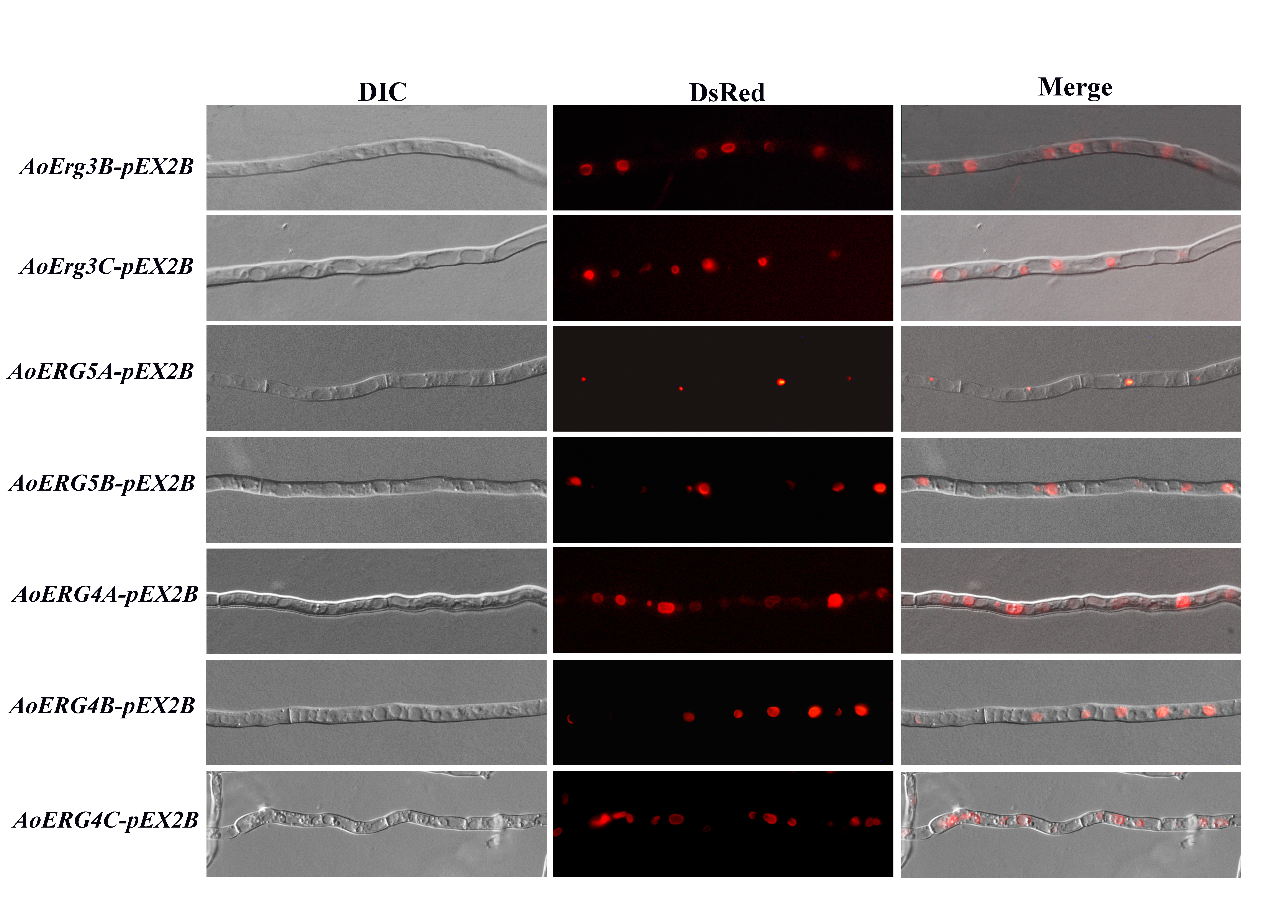


**Supplementary figure S6.** Fluorescence images of fluorescence-labeled ergosterol synthase not co-localized with certain organelles detected. Left to right: differential interference contrast (DIC), fluorescent images of GFP, fluorescent images of DsRed, merged images of GFP and DsRed, and merged images of DIC, GFP, and DsRed.


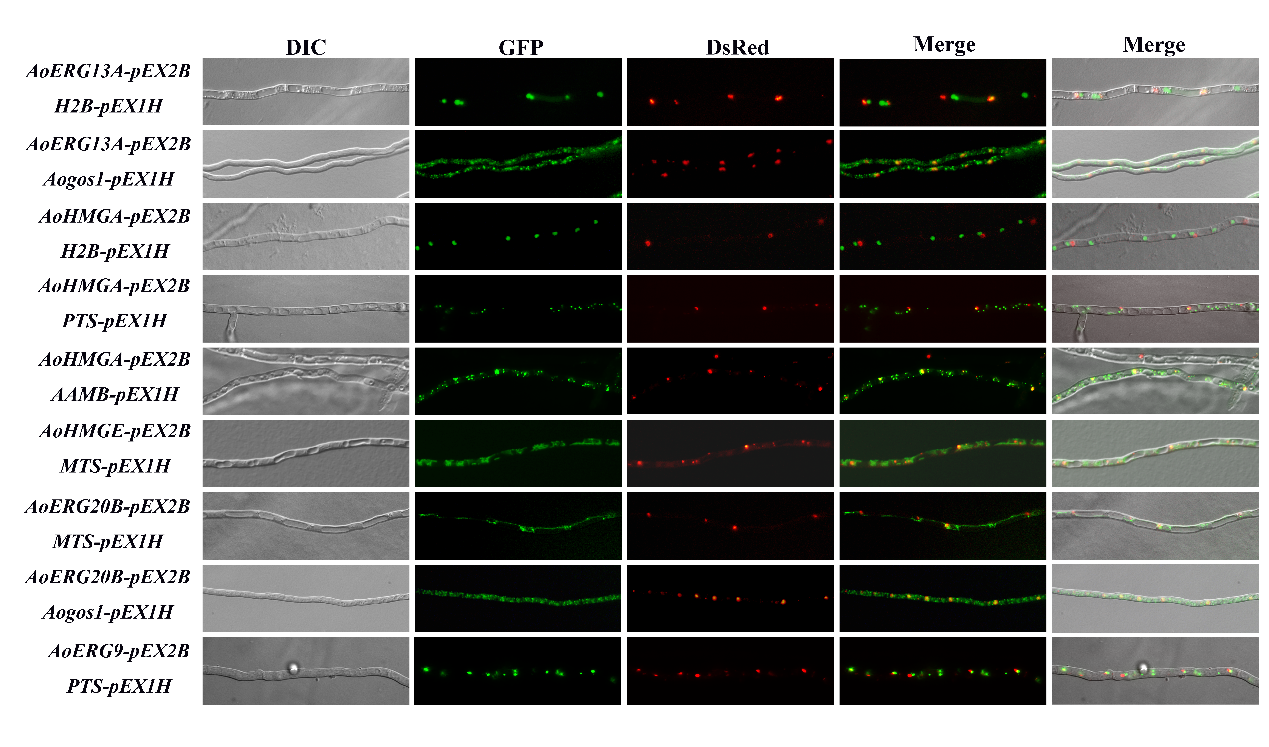


**Supplementary figure S7.** Subcellular localization of ergosterol biosynthetic enzymes and endoplasmic reticulum in *A. oryzae*. Left to right: differential interference contrast (DIC), fluorescent images of GFP, fluorescent images of DsRed, merged images of GFP and DsRed, and merged images of DIC, GFP, and DsRed.

**
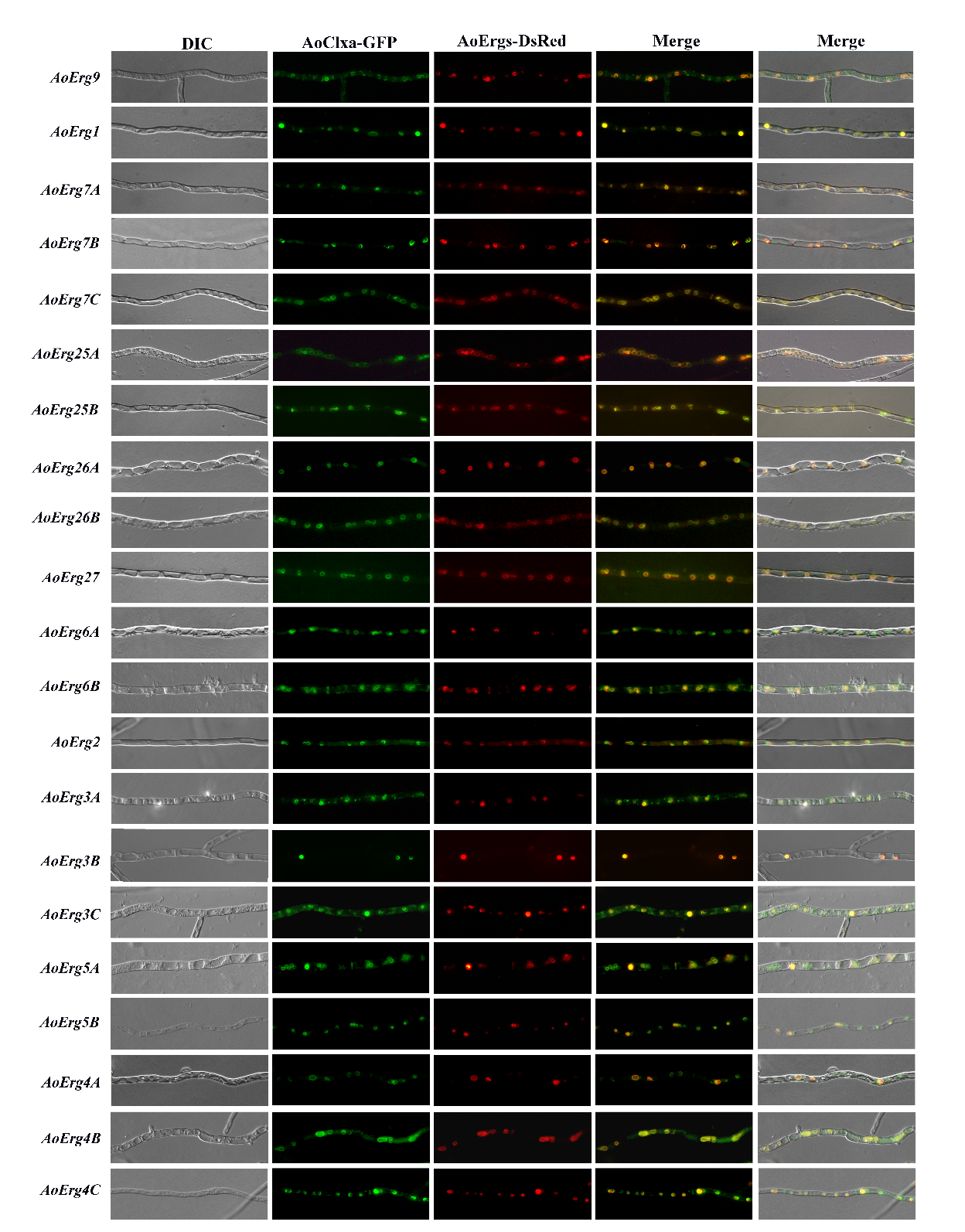
**

**Supplementary figure S8.** Phenotypes of ergosterol biosynthetic enzyme over-expressing strains. Colony morphologies of the control (AoCK), and overexpressing strains overexpression strains on the CD+His medium incubated at 30ºC for 72h.

**
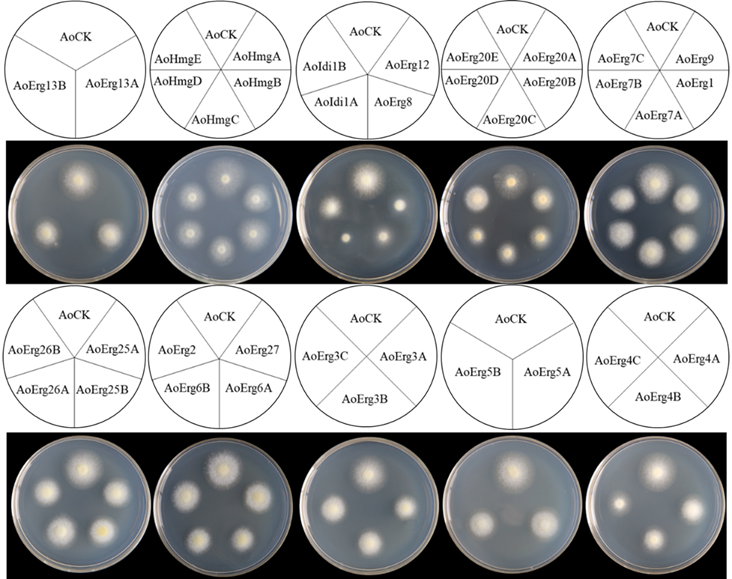
**

## Supplementary figure S9. Subcellular localization of Ao*Erg9/HmgB*,Ao*Erg1/HmgB*,Ao*Erg27/HmgB*;Ao*Erg7B/Erg1*,Ao*Erg27/Erg7B*,*AoErg7B/HmgB*. Left to right: differential interference contrast (DIC), fluorescent images of GFP, fluorescent images of DsRed, merged images of GFP and DsRed, and merged images of DIC, GFP, and DsRed.


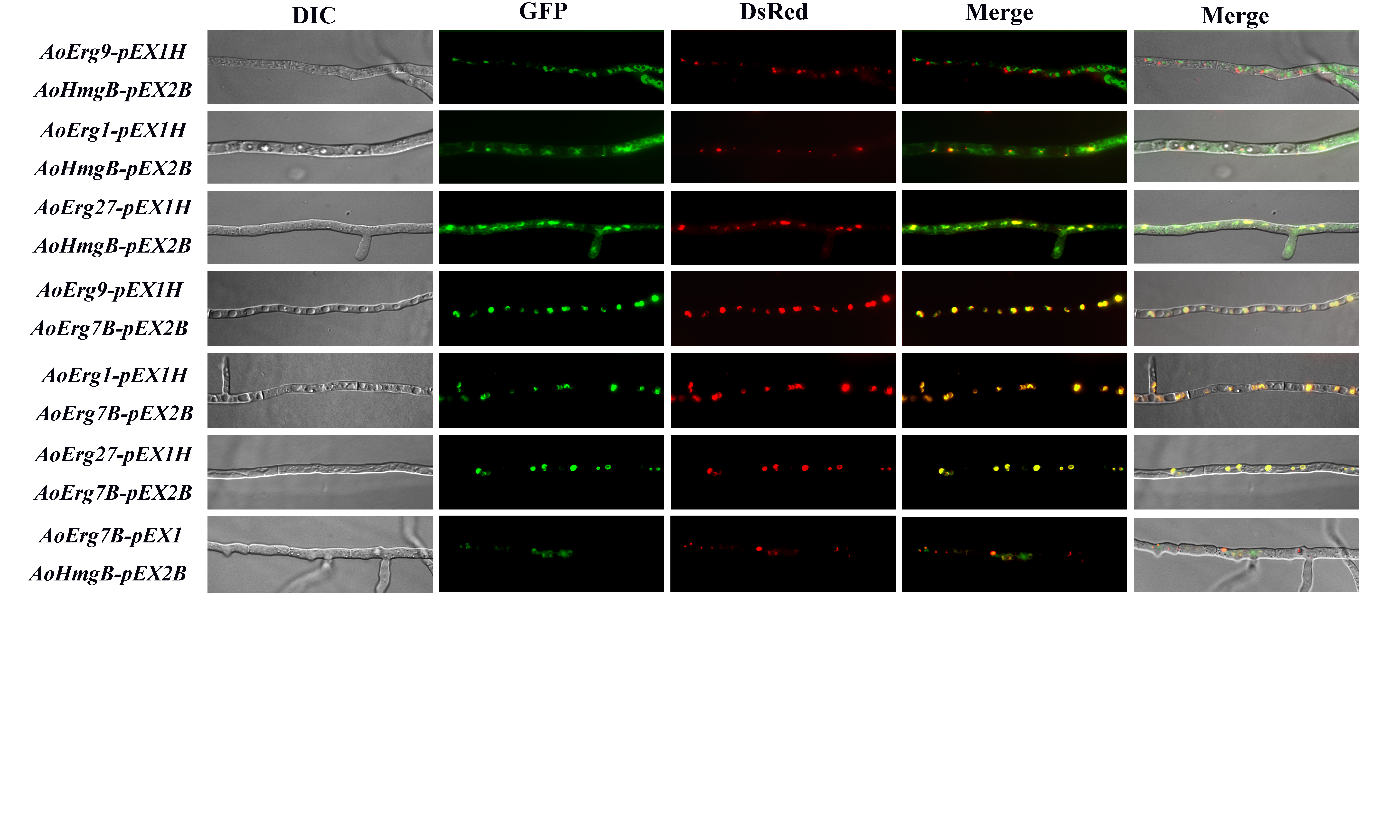


**Supplementary figure S10.** Fluorescent images of fluorescently-labeled organelle localization strains. Left to right: differential interference contrast (DIC), fluorescent images of GFP, merged images of DIC and GFP. H2B, MTS, AoCIxA, Aovam, AAMB, PTS, and Aogos1 represent the nucleus, mitochondria, endoplasmic reticulum, vesicles, lipid droplets, peroxisomes, and Golgi apparatus, respectively.

**
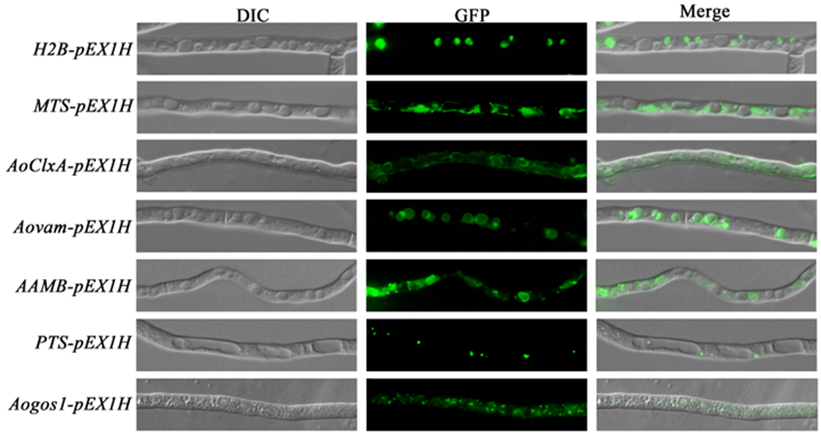
**

**Supplementary Table S1.** Expression analysis of ergosterol biosynthesis genes.

| **Gene** | ***A. oryzae* 3.042** | | **Different growth stage** -fpkm | | | **Function** |
| --- | --- | --- | --- | --- | --- | --- |
|  | **Paralogous** | **Protein ID** | 24h | 48h | 72h |  |
| *AoERG10* | 6 | EIT73661.1 | 313.85 | 318.98 | 270.18 | Acetyl-CoA acetyltransferase |
|  |  | EIT73496.1 | 68.21 | 67.51 | 66.03 |  |
|  |  | EIT79671.1 | 24.09 | 22.79 | 24.17 |  |
|  |  | EIT78121.1 | 30.90 | 26.70 | 23.41 |  |
|  |  | EIT78942.1 | 70.31 | 124.90 | 125.13 |  |
|  |  | EIT80840.1 | 371.42 | 576.01 | 571.69 |  |
| *AoERG13* | 2 | EIT76569.1 | 127.15 | 79.43 | 66.63 | Hydroxymethylglutaryl-CoA synthase |
|  |  | EIT75784.1 | 7.53 | 3.55 | 11.95 |  |
| *AoHMG1*/*2* | 5 | EIT73568.1 | 47.40 | 31.71 | 46.95 | Hydroxymethylglutaryl-CoA reductase |
|  |  | EIT78580.1 | 63.85 | 37.22 | 29.37 |  |
|  |  | EIT81748.1 | 0.47 | 0.38 | 0.47 |  |
|  |  | EIT83470.1 | 0.52 | 0.09 | 0.50 |  |
|  |  | EIT73603.1 | 0.53 | 1.11 | 0.85 |  |
| *AoERG12* | 1 | EIT78374.1 | 35.95 | 66.36 | 58.87 | Mevalonate kinase |
| *AoERG8* | 1 | EIT75656.1 | 119.80 | 88.41 | 91.84 | Phosphomevalonate kinase |
| *AoERG19* | 1 | EIT78501.1 | 142.46 | 150.12 | 125.19 | Diphosphomevalonate decarboxylase |
| *AoIDI1* | 2 | EIT75013.1 | 365.21 | 178.88 | 171.87 | Isopentenyl-diphosphate delta-isomerase |
|  |  | EIT75157.1 | 0.15 | 0.08 | 0.08 |  |
| *AoERG20* | 5 | EIT77142.1 | 111.60 | 94.94 | 101.58 | Bifunctional (2E,6E)-farnesyl diphosphate synthase/ dimethylallyltranstransferase |
|  |  | EIT82866.1 | 14.37 | 15.44 | 6.63 |  |
|  |  | EIT78828.1 | 0.00 | 0.00 | 0.00 |  |
|  |  | EIT74365.1 | 9.43 | 6.10 | 6.68 |  |
|  |  | EIT77275.1 | 0.02 | 0.18 | 0.23 |  |
| *AoEGR9* | 1 | EIT75610.1 | 93.43 | 92.12 | 121.15 | Squalene synthetase |
| *AoERG1* | 1 | EIT81755.1 | 119.93 | 65.89 | 83.33 | Squalene epoxidase |
| *AoERG7* | 3 | EIT83324.1 | 44.67 | 41.28 | 37.15 | Lanosterol synthase |
|  |  | EIT77904.1 | 34.87 | 79.37 | 97.29 |  |
|  |  | EIT79966.1 | 3.87 | 12.45 | 6.50 |  |
| *AoERG11* | 3 | EIT83124.1 | 121.95 | 97.74 | 95.86 | Sterol14-demethylase |
|  |  | EIT73378.1 | 88.93 | 26.94 | 35.65 |  |
|  |  | EIT72345.1 | 0.14 | 0.07 | 0.14 |  |
| *AoERG24* | 2 | EIT78405.1 | 32.72 | 22.79 | 21.28 | Sterol C-14 reductase |
|  |  | EIT72491.1 | 23.31 | 9.57 | 10.19 |  |
| *AoERG25* | 2 | EIT77737.1 | 226.29 | 134.15 | 170.86 | Sterol C-4 methyloxidases |
|  |  | EIT75499.1 | 105.93 | 3.75 | 4.91 |  |
| *AoERG26* | 2 | EIT79126.1 | 1.21 | 3.75 | 1.94 | Sterol C-4 decarboxylases |
|  |  | EIT82518.1 | 8.25 | 1.22 | 3.60 |  |
| *AoERG27* | 1 | EIT80831.1 | 21.81 | 15.62 | 17.11 | Sterol 3-keto reductases |
| *AoERG6* | 2 | EIT83284.1 | 102.23 | 58.84 | 67.62 | Sterol C-24 methyltransferases |
|  |  | EIT80311.1 | 0.63 | 0.38 | 0.48 |  |
| *AoERG2* | 1 | EIT81783.1 | 59.53 | 37.78 | 36.45 | Sterol C-8 isomerases |
| *AoERG3* | 3 | EIT79919.1 | 34.03 | 0.68 | 1.09 | Sterol C-5 desaturases |
|  |  | EIT80397.1 | 73.05 | 37.50 | 40.46 |  |
|  |  | EIT73679.1 | 23.38 | 14.99 | 25.16 |  |
| *AoERG5* | 2 | EIT82696.1 | 26.18 | 30.21 | 26.24 | Sterol C-22 desaturases |
|  |  | EIT73398.1 | 148.04 | 45.07 | 59.15 |  |
| *AoERG4* | 3 | EIT80299.1 | 17.91 | 13.22 | 15.48 | Sterol C-24 reductases |
|  |  | EIT77041.1 | 26.91 | 27.89 | 22.69 |  |
|  |  | EIT74004.1 | 0.39 | 1.01 | 1.22 |  |

**Supplementary Table S2.** The fatty acid content in ergosterol biosynthetic enzyme overexpression strains.

**Supplementary Table S3.** The number of DEGs compared with control.

| DEG set | Number of differentially expressed genes | Number of up-regulated genes | Down-regulation of the number of genes |
| --- | --- | --- | --- |
| AoHmgB vs CK | 1806 | 361 | 1445 |
| Erg7B vs CK | 5005 | 2556 | 2449 |
| AoHmgB vs AoErg7B | 4411 | 2796 | 1615 |

**Supplementary Table S4.** DEGs in AoHmgB overexpressed strain compared with control.

| Classifications | Functional classification | Total | Up | Down | |
| --- | --- | --- | --- | --- | --- |
|  | toxin biosynthetic process | 8 | 8 | 0 |  |
|  | toxin metabolic process | 8 | 8 | 0 | |
|  | mycotoxin metabolic process | 8 | 8 | 0 | |
| BP | mycotoxin biosynthetic process | 8 | 8 | 0 | |
|  | secondary metabolic process | 8 | 8 | 0 | |
|  | secondary metabolite biosynthetic process | 8 | 8 | 0 | |
|  | lipid catabolic process | 9 | 7 | 2 | |
|  | coenzyme binding | 90 | 60 | 30 | |
|  | oxidoreductase activity, acting on paired donors, with incorporation or reduction of molecular oxygen | 48 | 45 | 3 | |
|  | heme binding | 44 | 40 | 4 | |
| MF | tetrapyrrole binding | 44 | 40 | 4 | |
|  | iron ion binding | 45 | 39 | 6 | |
|  | flavin adenine dinucleotide binding | 44 | 35 | 9 | |
|  | O-methyltransferase activity | 11 | 11 | 0 | |
|  | vitamin binding | 22 | 8 | 14 | |

**Supplementary Table S5.** DEGs in AoErg7B overexpressed strain compared with control.

| classifications | Functional classification | Total | Up | Down | |
| --- | --- | --- | --- | --- | --- |
| BP | carbohydrate metabolic process | 139 | 65 | 74 |  |
| CC | ribosome | 60 | 60 | 0 |  |
|  | ribonucleoprotein complex | 62 | 62 | 0 |  |
|  | transporter activity | 219 | 93 | 126 |  |
|  | transmembrane transporter activity | 216 | 91 | 125 |  |
|  | structural constituent of ribosome | 61 | 61 | 0 |  |
|  | iron ion binding | 102 | 45 | 57 |  |
| MF | coenzyme binding | 198 | 97 | 101 |  |
|  | heme binding | 88 | 40 | 48 |  |
|  | tetrapyrrole binding | 88 | 40 | 48 |  |
|  | flavin adenine dinucleotide binding | 105 | 51 | 54 |  |
|  | structural molecule activity | 62 | 61 | 1 |  |
|  | oxidoreductase activity, acting on paired donors, with incorporation or reduction of molecular oxygen | 94 | 43 | 51 |  |

**Supplementary Table S6.** DEGs of ergosterol synthesis-related genes.

| Function classes | Fold changes in gene expression | | |
| --- | --- | --- | --- |
|  | AoHmgB vs CK | AoErg7B vs CK | AoHmgB vs AoErg7B |
| *AoErg1*（EIT81755.1） | ND | 3.37 | -3.30 |
| *AoErg7B* (EIT77904.1) | ND | 2.65 | -2.19 |
| *AoErg7C*（EIT79966.1） | 3.64 | -3.27 | 6.93 |
| *AoErg11A* (EIT83124.1) | ND | 1.92 | -1.72 |
| *AoErg11B* (EIT73378.1) | 2.68 | 3.57 | ND |
| *AoErg24A* (EIT78405.1) | ND | 3.01 | -2.41 |
| *AoErg24B*（EIT72491.1） | 2.32 | 3.74 | -1.39 |
| *AoErg25A* (EIT77737.1) | 1.63 | 4.15 | -2.50 |
| *AoErg25B*（EIT75499.1） | 3.58 | 9.62 | -6.02 |
| *AoErg26B* (EIT82518.1) | ND | 3.50 | -3.25 |
| *AoErg6A* (EIT83284.1) | ND | 1.45 | -1.14 |
| *AoErg6B* (EIT80311.1） | 4.25 | 9.36 | -5.10 |
| *AoErg2* (EIT81783.1) | ND | 1.73 | ND |
| *AoErg3B* (EIT80397.1) | ND | 3.00 | -2.27 |
| *AoErg3C* ([EIT73679.1](http://www.ncbi.nlm.nih.gov/protein/391864383?report=genbank&log$=prottop&blast_rank=3&RID=WWC7R6HF014)） | 1.30 | 3.99 | -2.67 |
| *AoErg4A* (EIT80299.1) | ND | 1.40 | ND |
| *AoErg4B* (EIT77041.1) | ND | -3.62 | 3.16 |

**Supplementary Table S7.** Primer sequences for gene over-expression.

**Supplementary Table S8.** Primer sequences for combined over-expression.
